# Association of Circulating Cathepsin S and Cardiovascular Disease Among Patients With Type 2 Diabetes: A Cross-Sectional Community-Based Study

**DOI:** 10.3389/fendo.2021.615913

**Published:** 2021-03-05

**Authors:** Yu Jing, Jie Shi, Bin Lu, Weiwei Zhang, Yehong Yang, Jie Wen, Renming Hu, Zhen Yang, Xuanchun Wang

**Affiliations:** ^1^ Department of Endocrinology, Huashan Hospital, Fudan University, Shanghai, China; ^2^ Department of Endocrinology, Xinhua Hospital, Shanghai Jiaotong University School of Medicine, Shanghai, China

**Keywords:** cathepsin S, cardiovascular disease, type 2 diabetes, adipokine, diagnostic biomarker

## Abstract

**Background:**

Cathepsin S, as an adipokine, was reported to play a critical role in various disease, including atherosclerosis and diabetes. The present study aims to elucidate the relationship between circulating cathepsin S and cardiovascular disease (CVD) in patients with type 2 diabetes.

**Methods:**

A total of 339 type 2 diabetes individuals were enrolled in this cross-sectional community-based study. Basic information, medical and laboratory data were collected. Serum cathepsin S levels were assessed by ELISA.

**Results:**

Compared to the CVD (−) group, levels of serum cathepsin S were significantly higher in the CVD (+) group, with the median 23.68 ng/ml (18.54–28.02) and 26.81 ng/ml (21.19–37.69) respectively (*P* < 0.001). Moreover, patients with acute coronary syndrome (ACS) had substantially higher levels of serum cathepsin S than those with stable angina pectoris (SAP), with the median 34.65 ng/ml (24.33–42.83) and 25.52 ng/ml (20.53–31.47) respectively (*P* < 0.01). The spearman correlation analysis showed that circulating cathepsin S was correlated with several cardiovascular risk factors. The univariate and multivariate logistic regression analysis revealed that circulating cathepsin S was an independent risk factor for CVD (all *P* < 0.001) after adjustment for potential confounders. Restricted cubic spline analysis showed circulating cathepsin S had a linearity association with CVD. In addition, receiver operating characteristic (ROC) curve analysis demonstrated that the area under curve (AUC) values of cathepsin S was 0.80 (95% CI: 0.75–0.84, *P* < 0.001), with the optimal cutoff value of cathepsin 26.28 ng/ml.

**Conclusion:**

Circulating cathepsin S was significantly higher in the CVD (+) group than that in the CVD (−) one among type 2 diabetes. The increased serum cathepsin S levels were associated with increased risks of CVD, even after adjusting for potential confounders. Thus, cathepsin S might be a potential diagnostic biomarker for CVD.

## Introduction

Cardiovascular disease (CVD) is the major complication and leading cause of morbidity and mortality in patients with type 2 diabetes (1). It is particularly important to detect and prevent the development of cardiovascular complications among patients with type 2 diabetes.

Cathepsin S, which belongs to the cysteine proteinase family (2), is one of the most potent mammalian elastases that can degrade many extracellular elements, such as elastin, fibronectin, laminin, and collagens (3). Previous studies have revealed that cathepsin S plays a critical role in various diseases, including atherosclerosis (4), abdominal aortic aneurysm (5), cancer (6), obesity (7) and type 2 diabetes (8). Liu et al. reported that there is a significant increase of circulating cathepsin S level in both diabetes and atherosclerosis (9). And Li et al. discovered an increased level of cathepsin S at both the mRNA and protein levels in non-obese diabetic mouse model (10). Meanwhile, it was found that cathepsin S abounds in human atherosclerotic lesions, in macrophages, smooth muscle cells (SMCs), and endothelial cells (11). Several clinical studies suggested that cathepsin S may be involved in the destabilization and rupture of atherosclerotic plaques (12–14). Thus, the aforementioned studies indicated that cathepsin S may serves as a biomarker for disease such as diabetes and atherosclerosis.

However, little is known about the direct relationship between circulating cathepsin S and CVD in the context of type 2 diabetes. Hence, we undertook this study and investigated the cross-sectional community-based association between circulating cathepsin S and CVD in patients with type 2 diabetes.

## Materials and Methods

### Study Population

This cross-sectional study was conducted in twenty residential areas of Shanghai from February 2004 to July 2004. A total of 339 patients with type 2 diabetes were enrolled. All subjects had written informed consent to participate in the study. Inclusion criteria included established T2DM diagnosis according to the 1999 World Health Organization diagnostic criteria (fasting plasma glucose ≥ 7.0 mmol/L and/or 2 h plasma glucose ≥ 11.1 mmol/L). Exclusion criteria were type 1 diabetes, gestational diabetes, or diabetes induced by steroid use or other endocrine diseases. Based on whether subjects have cardiovascular disease (including established coronary, cerebrovascular and peripheral vascular disease) detected by hospital medical records or a thorough physical examination by trained physicians. These subjects were divided into CVD (−) group (n = 175) and CVD (+) group (n = 164). Moreover, patients with coronary artery disease (CAD) were further sub-divided into stable angina pectoris (SAP) group (n=43) and acute coronary syndrome (ACS) group (n=61) according to their symptoms and clinical examinations. The study protocol was approved by the Institutional Review Broad of Huashan Hospital, Fudan University.

### Data Collection

A standardized questionnaire was applied by trained physicians to collect basic information, including age, sex, smoking (yes/no), alcohol consumption (yes/no) as well as medical history including diabetes, hypertension and cardiovascular disease. Anthropometric measurements including height, weight, waist circumference, hip circumference, blood pressure, and BMI were taken by trained physicians using standard protocols in duplicate base. Waist circumference was measured with a non-elastic tape held midpoint between the lower rib margin and the top of iliac crest at the end of the expiration phase. Hip circumference was measured as the maximum circumference at the level of the buttocks. Blood pressure was recorded 3 min apart on the non-dominant arm, with an appropriate-sized cuff after the patient had been sitting for 5 min. BMI was calculated as the weight in kilograms divided by the square of height in meters.

### Biochemical Measurements

Peripheral venous blood samples were collected after an overnight fast for at least 10 h. Blood glucose, serum insulin, total cholesterol (TC), triglycerides (TG), low‐density lipoprotein cholesterol (LDL‐C), high‐density lipoprotein cholesterol (HDL‐C), blood urea nitrogen (BUN), serum creatine and uric acid were measured by using an automatic analyser (Hitachi 7080; Tokyo, Japan). Glycated haemoglobin (HbA1c) was determined by high pressure liquid chromatography using an analyser (HLC-723G7, Tosoh Corporation, Japan). eGFR was assessed with the CKD-EPI equation (15). Insulin resistance was assessed using the homeostasis model assessment index‐insulin resistance (HOMA‐IR).

### Measurements of Cathepsin S

Serum levels of cathepsin S were measured in duplicate by ELISA (human cathepsin S (total), DY1183; R&D Systems, Minneapolis, MN, USA) according to the recommendation by the manufacturer. The intra-assay coefficient of variation was 7.5% to 9.4%, and the inter-assay coefficient of variation was 6.3% to 10.2%.

### Definition of Cardiovascular Disease (CVD)

The presence of CVD was evaluated as follows. A detailed medical history was recorded by trained physicians, including previous or current coronary artery disease (asymptomatic chronic coronary syndrome, angina, myocardial infarction, surgical or percutaneous coronary revascularization procedure, or at least one major coronary artery stenosis ≥ 50% diagnosed by coronary angiography), cerebrovascular disease (ischemic stroke, hemorrhagic stroke, transient ischemic attacks, carotid revascularization procedure, or carotid stenosis ≥ 70% evidenced by echo-Doppler scanning), and peripheral vascular disease (intermittent claudication with a documented low ankle-brachial index (ABI **<** 0.9), rest pain, as evaluated by echo-Doppler scanning, obstructive disease on femoral angiography, or lower limb revascularization procedure). The presence of CVD was confirmed by reviewing hospital medical records and undertaking a thorough physical examination by trained physicians. Vascular laboratory data, such as electrocardiogram and echo-Doppler scanning of the carotid and lower limb arteries were also performed for all participants. Data on CVD were collected for all the patients.

Meanwhile, patients with coronary artery disease (CAD) were further sub-divided into stable angina pectoris (SAP) group and acute coronary syndrome (ACS) group by symptoms and clinical examinations. SAP was diagnosed as an invariable exertional chest pain lasting 3 to 5 min, and every time, the pain is of the same location, same degree of exertion and excitation provocation, which can be relieved by rest or nitroglycerin. ACS including unstable angina pectoris (UAP) and acute myocardial infarction (AMI). UAP was diagnosed by typical chest pain at rest. And the patients had depressed ST ≥ 0.1 mV, and/or T-wave inversion on electrocardiogram but with normal creatine kinase-MB level. AMI was diagnosed by elevated cardiac biomarkers (at least one positive biomarker: creatine kinase-MB, or troponin T) and electrocardiogram showed new ischemia (new ST-T change or new left bundle branch block), besides, patients had a prolonged chest pain medical history (16).

In addition, the following definitions were used for cardiovascular risk factors: Hypertension was defined as a systolic blood pressure > 140 mm Hg, a diastolic blood pressure > 90 mm Hg, and/or having received treatment for hypertension. Hyperlipidemia was defined as total cholesterol > 200 mg/dL and/or having received treatment with statins. Hyperglycemia was defined as a serum glucose > 140 mg/dL and/or having received treatment with anti-hyperglycemic medication. The history of smoking or alcohol was defined as never, current (smoking or consuming alcohol regularly in the past 6 months), or ever (cessation of smoking or alcohol consumption for more than 6 months).

### Statistical Analysis

The Kolmogorov-Smirnov test was used to evaluate the normality and variance uniformity of the data. Continuous variables with normal distribution were presented as means ± SD, while variables with a skewed distribution were expressed as median (interquartile range) and log-transformed to approximate normality before analysis. Categorical variables were shown as frequencies and percentages. Differences between groups were examined by Student’s t-tests (for continuous parametric variables), Mann–Whitney U tests (for continuous nonparametric variables) and Chi-squared tests (for categorical variables). Spearman correlation was used to estimate the relationship between circulating cathepsin S and other clinical indexes. Univariate and multivariate logistic regression analysis were used to estimate the association between independent variables and CVD (with or without). Variables entered in the multivariate model were chosen according to the clinical and statistical significance. Any variable having significant univariate test (*P* **<** 0.05) was selected as a candidate for the multivariate analysis. Restricted cubic spline (RCS) was constructed to examine the potential relationship between circulating cathepsin S and the risk of CVD incidence. Receiver operating characteristic (ROC) curve analysis was further utilized to check the diagnostic effectiveness of cathepsin S. All statistical analyses were performed using IBM SPSS Version 25 (SPSS Statistics V25, IBM Corporation, Somers, New York), R version 3.5.2 (R Foundation for Statistical Computing) and Prism 8 software. A two-sided *P* **<** 0.05 was considered statistically significant in all analyses.

## Results

### Patients’ Characteristics

A total of 339 type 2 diabetes patients were enrolled in this study, including 130 males and 209 females. The mean age was 68.11 ± 10.04 years and the mean course of diabetes was 8.69 ± 7.44 years with an average HbA1c 6.70% (6.08–8.00) and the mean BMI of 24.76 ± 3.51kg/m^2^.

We divided these patients into CVD (−) group (n = 175) and CVD (+) group (n = 164) based on their diagnosis of CVD. Among CVD (+) group, 44 patients with CAD alone, 30 with cerebral vascular disease alone, 18 with peripheral vascular disease alone, 32 with both CAD and cerebral vascular disease, 20 with both CAD and peripheral vascular disease, 12 with both cerebral vascular disease and peripheral vascular disease, 8 with both CAD, cerebral vascular disease and peripheral vascular disease. All the patients’ clinical and biochemical characteristics are summarized in **Table 1**. T2DM patients in the study with CVD diagnosed were more likely to be alcohol drinkers, and had comorbidities including hypertension (all *P* < 0.05). Besides, levels of 2 h insulin (*P* < 0.001), fasting C peptide (*P* < 0.05), 2 h C peptide (*P* < 0.001), TG (*P* < 0.01), LDL-C (*P* < 0.01), serum uric acid (*P* < 0.01) and cathepsin S (*P* < 0.001) were substantially higher in the CVD (+) group compared with the CVD (−) group. Moreover, patients in the CVD (+) group tended to have higher rates of anti-hypertensive, anti-platelet and lipid lowering drug usages. However, there were no statistical difference in age, sex, smoking status, duration of type 2 diabetes, body mass index (BMI), waist circumference (WC), waist–hip ratio (WHR), systolic blood pressure (SBP), diastolic blood pressure (DBP), fasting blood glucose (FBG), postprandial blood glucose (PBG), fasting insulin, HOMA-IR, HbA1c, TC, HDL-C, BUN, serum creatine, eGFR and anti-diabetic drug usages between two groups.

**Table 1:** Clinical and biochemical characteristics of the study subjects.

Characteristics	Overall (N = 339)	CVD (−) (n = 175)	CVD (+) (n = 164)	*P* value
**Anthropometric parameters**				
Age (years)	68.11 ± 10.04	67.61 ± 10.61	68.63 ± 9.41	0.140
Sex (male/female)	130/209	73/102	57/107	0.188
Smoking, n (%)	66(19.5%)	37(21.1%)	29(17.7%)	0.421
Alcohol, n (%)	108(31.9%)	52(29.7%)	68(41.5%)	0.024^*^
Hypertension, n (%)	267(78.8%)	126(72%)	141(86%)	0.002^**^
Duration of diabetes (years)	8.69 ± 7.44	8.79 ± 7.49	8.59 ± 7.42	0.230
BMI (kg/m^2^)	24.76 ± 3.51	23.79 ± 3.03	25.77 ± 3.70	0.121
WC (cm)	86.10 ± 8.73	86.11 ± 8.46	86.09 ± 9.03	0.983
WHR	0.88 ± 0.06	0.88 ± 0.06	0.88 ± 0.06	0.529
SBP (mm Hg)	140 (130–152)	140 (130–150)	140 (130–156)	0.065
DBP (mm Hg)	80 (70–90)	80 (70–85.5)	80 (75.5–90)	0.060
**Metabolic parameters**				
FBG (mmol/L)	7.80 (6.80–9.90)	8.00 (6.70–9.85)	7.80 (6.88–9.90)	0.791
PBG (mmol/L)	13.90 (10.80–18.50)	13.80 (10.70–16.90)	14.05 (10.90–20.10)	0.078
Fasting insulin (pmol/L)	12.25 (7.46–18.17)	11.63 (6.12–17.99)	12.55 (8.34–18.22)	0.152
2 h insulin (pmol/L)	33.71 (19.47–51.99)	27.18 (16.08–39.50)	41.07 (26.08–61.35)	0.000^***^
Fasting C peptide (ng/ml)	3.42 (2.71–4.57)	3.32 (2.62–4.28)	3.66 (2.85–4.82)	0.041^*^
2 h C peptide (ng/ml)	8.31 (5.74–11.51)	7.14 (5.10–9.89)	9.60 (6.65–12.93)	0.000^***^
HOMA-IR	4.50 (2.51–7.28)	4.28 (2.13–7.26)	4.68 (2.70–7.27)	0.228
HbA1c %	6.70 (6.08–8.00)	6.70 (5.90–8.00)	6.70 (6.10–7.95)	0.395
TC (mmol/L)	5.32 (4.61–6.01)	5.14 (4.61–5.83)	5.51 (4.59–6.12)	0.063
TG (mmol/L)	1.65 (1.18–2.37)	1.54 (1.04–2.12)	1.74 (1.32–2.53)	0.003^**^
LDL-C (mmol/L)	3.00 (2.45–3.50)	2.80 (2.40–3.40)	3.10 (2.60–3.60)	0.006^**^
HDL-C (mmol/L)	1.30 (1.00–1.50)	1.30 (1.05–1.55)	1.25 (1.00–1.50)	0.275
BUN (mmol/L)	6.10 (5.10–7.10)	6.00 (5.00–7.10)	6.10 (5.10–6.93)	0.949
Scr (μmol/L)	64.00 (54.00–78.00)	63.00 (53.00–78.00)	65.00 (55.00–78.00)	0.457
Serum uric acid (μmol/L)	280 (230–330)	270 (220–320)	290 (240–350)	0.006^**^
eGFR (ml·min^−1^ 1.73m^−2^)	120.52 ± 28.50	123.64 ± 28.38	117.25 ± 28.34	0.773
Cathepsin S (ng/ml)	25.22 (19.76–32.65)	23.68 (18.54–28.02)	26.81 (21.19–37.69)	0.000^***^
**Medications**				
ARBs or ACEIs	101 (29.8%)	35 (20%)	66 (40.2%)	0.000^***^
CCBs	88 (26.0%)	17 (9.7%)	71 (43.3%)	0.000^***^
β-blocker	26 (7.7%)	9 (5.1%)	17 (10.4%)	0.071
Aspirin	201 (59.3%)	37 (21.1%)	164 (100%)	0.000^***^
Lipid lowering drugs	150 (44.2%)	58 (33.1%)	92 (56.1%)	0.000^***^
Anti-diabetic drugs	260 (76.7%)	133 (76%)	127 (77.4%)	0.754
**Cardiovascular disease**				
CAD alone	44 (13.0%)	0	44 (26.8%)	
Cerebral vascular disease alone	30 (8.8%)	0	30 (18.3%)	
Peripheral vascular disease alone	18 (5.3%)	0	18 (11.0%)	
Both CAD and cerebral vascular disease	32 (9.4%)	0	32 (19.5%)	
Both CAD and peripheral vascular disease	20 (5.9%)	0	20 (12.2%)	
Both cerebral vascular disease and peripheral vascular disease	12 (3.5%)	0	12 (7.3%)	
Both CAD, cerebral vascular disease and peripheral vascular disease	8 (2.4%)	0	8 (4.9%)	

Data are reported as mean ± SD, median (interquartile range), or number (proportion %), P < 0.05 (*), P < 0.01 (**), P < 0.001 (***).

### Difference in Serum Cathepsin S Between the Two Groups

**Figure 1:**
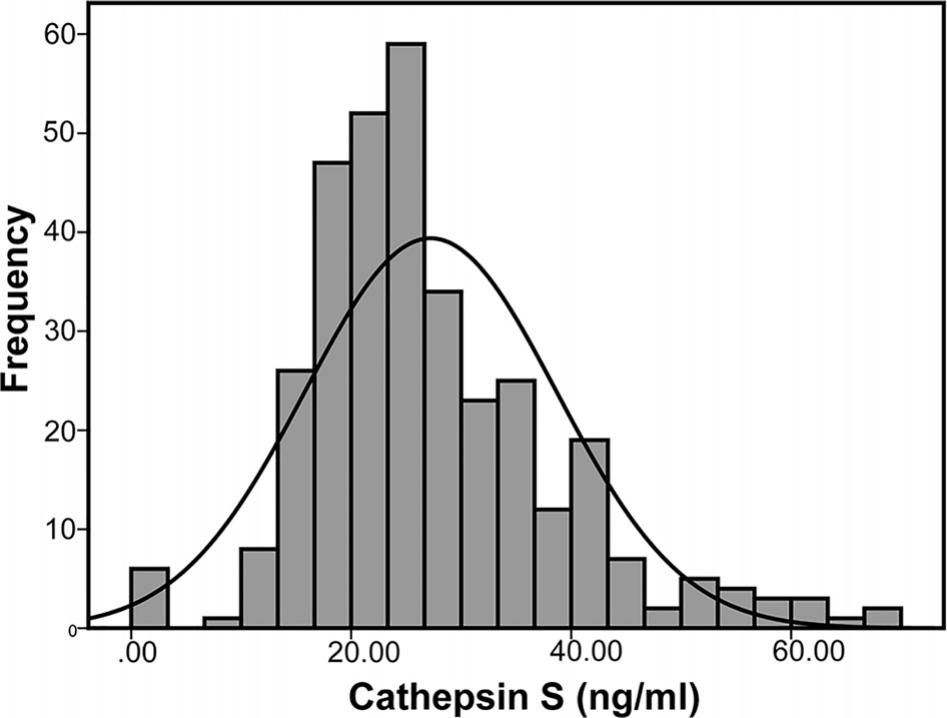
Distribution of serum cathepsin S levels in the patients.

**Figure 2:**
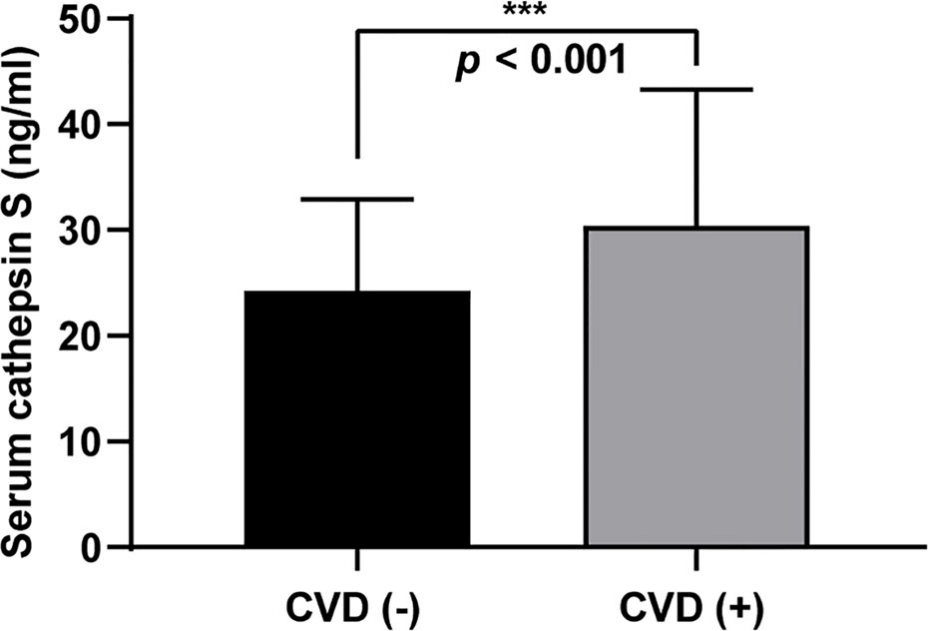
Circulating cathepsin S levels in the CVD (−) and CVD (+) group. Data are reported as median (interquartile range), *P* < 0.001 (***).

As presented in **Figure 1**, the median value of serum cathepsin S in the study population was 25.22 ng/ml (19.76–32.65). And the CVD (+) group had significantly higher cathepsin S levels than the CVD (−) group, with the median level 26.81 ng/ml (21.19–37.69) and 23.68 ng/ml (18.54–28.02) respectively. (*P* **<** 0.001, **Figure 2**).

### Characteristics of the Patients in SAP and AMI Groups

Since most patients suffered from CAD (including CAD alone, both CAD and cerebral vascular disease, both CAD and peripheral vascular disease, both CAD, cerebral vascular disease and peripheral vascular disease, n=104), we then sub-divided these patients into stable angina pectoris (SAP) group (n=43) and acute coronary syndrome (ACS) group (n=61). As shown in **Table 2**, patients with acute coronary syndrome (ACS) had substantially higher levels of serum cathepsin S than those with stable angina pectoris (SAP), with the median 34.65 ng/ml (24.33–42.83) and 25.52 ng/ml (20.53–31.47), respectively (*P* < 0.01). And patients with ACS had significantly higher levels of BMI (*P* < 0.05), DBP (*P* < 0.001), PBG (*P* < 0.05), 2 h insulin (*P* < 0.05) and cathepsin S (*P* < 0.01) than those with SAP. However, there were no statistical difference in other anthropometric parameters, metabolic parameters, and medications between two groups.

**Table 2:** Clinical and biochemical characteristics of the CAD subjects (SAP group and ACS group).

Characteristics	SAP (n=43)	ACS (n=61)	*P* value
**Anthropometric parameters**			
Age (years)	70.05 ± 8.12	71.19 ± 9.00	0.503
Sex (male/female)	13/30	22/39	0.535
Smoking, n (%)	10(23.3%)	15(24.6%)	0.875
Alcohol, n (%)	23(53.5%)	32(52.5%)	0.918
Hypertension, n (%)	36(83.7%)	52(85.2%)	0.832
Duration of diabetes (years)	9.00 ± 7.26	8.53 ± 7.80	0.762
BMI (kg/m^2^)	24.62 ± 3.36	26.20 ± 3.37	0.020^*^
WC (cm)	87.30 ± 9.45	85.30 ± 8.44	0.259
WHR	0.87 ± 0.06	0.87 ± 0.06	0.996
SBP (mm Hg)	140 (125–155)	140 (135–150)	0.307
DBP (mm Hg)	80 (70–80)	84 (80–90)	0.000^***^
**Metabolic parameters**			
FBG (mmol/L)	7.40 (6.70–8.90)	7.70 (7.00–10.05)	0.213
PBG (mmol/L)	13.00 (10.00–16.80)	16.00 (11.25–21.40)	0.048^*^
Fasting insulin (pmol/L)	11.11 (7.31–16.98)	14.07 (7.01–22.38)	0.176
2 h insulin (pmol/L)	35.45 (18.25–50.84)	43.52 (28.46–66.02)	0.036^*^
Fasting C peptide (ng/ml)	3.52 (2.67–4.54)	4.07 (2.96–6.18)	0.102
2 h C peptide (ng/ml)	7.51 (6.06–12.68)	9.45 (6.95–12.99)	0.196
HOMA-IR	4.18 (2.59–5.83)	4.86 (2.27–7.76)	0.329
HbA1c %	6.60 (6.23–7.90)	6.80 (6.10–7.50)	0.728
TC (mmol/L)	5.25 (4.59–5.87)	5.85 (4.65–6.30)	0.104
TG (mmol/L)	1.69 (1.38–2.63)	1.72 (1.22–2.65)	0.830
LDL-C (mmol/L)	3.00 (2.40–3.40)	3.30 (2.70–3.80)	0.154
HDL-C (mmol/L)	1.40 (1.10–1.60)	1.30 (1.10–1.70)	0.874
BUN (mmol/L)	6.10 (4.90–6.90)	6.10 (5.15–7.20)	0.584
Scr (μmol/L)	65.00 (55.00–83.00)	65.00 (55.00–77.00)	0.567
Serum uric acid (μmol/L)	280 (230–340)	270 (230–340)	0.680
eGFR (ml·min^−1^ 1.73m^−2^)	112.61 ± 25.31	119.42 ± 26.23	0.189
Cathepsin S (ng/ml)	25.52 (20.53–31.47)	34.65 (24.33–42.83)	0.001^**^
**Medications**			
ARBs or ACEIs	20 (46.5%)	26 (42.6%)	0.694
CCBs	17 (39.5%)	27 (44.3%)	0.631
β-blocker	5 (11.6%)	7 (11.5%)	0.981
Aspirin	43 (100%)	61 (100%)	
Lipid lowering drugs	34 (79.1%)	50 (82.0%)	0.712
Anti-diabetic drugs	33 (76.7%)	50 (82.0%)	0.513

Data are reported as mean ± SD, median (interquartile range), or number (proportion %), P < 0.05 (*), P < 0.01 (**), P < 0.001 (***).

### Correlation Between Serum Cathepsin S and Other Clinical Parameters

The Spearman correlation analysis demonstrated that among various metabolic features, cathepsin S was positively correlated with BMI, WHR, SBP, DBP, 2 h insulin, BUN and TG (all *P* < 0.05, **Table 3**).

**Table 3:** Correlation of circulating cathepsin S with clinical and biological parameters.

Variables	cathepsin S	
	r	*p* value
Age	−0.016	0.771
Sex	−0.026	0.635
Smoking	0.013	0.818
Alcohol	−0.036	0.507
Hypertension	0.107	0.051
Duration of diabetes	−0.001	0.981
BMI	0.143	0.009^**^
WC	0.021	0.696
WHR	0.126	0.021^*^
SBP	0.132	0.016^*^
DBP	0.216	0.000^***^
FBG	0.078	0.154
PBG	0.100	0.069
HbA1c	0.022	0.692
Fasting insulin	0.067	0.221
2 h insulin	0.129	0.019^*^
Fasting C peptide	0.039	0.469
2 h C peptide	0.102	0.063
HOMA-IR	0.106	0.051
BUN	0.118	0.030^*^
Scr	0.023	0.668
Serum uric acid	−0.013	0.814
eGFR	−0.031	0.575
TC	−0.021	0.695
TG	0.108	0.046^*^
LDL-C	−0.048	0.381
HDL-C	−0.018	0.737

P < 0.05 (*), P < 0.01 (**), P < 0.001 (***).

### Association Between Cathepsin S and CVD

To identify the associated factors with CVD, univariate and multivariate logistic regression analysis were used to fit the models between CVD and other independent variables. The univariate logistic regression analysis showed that clinical factors including alcohol consumption, history of hypertension, BMI, DBP, PBG, 2 h insulin, 2 h C peptide, serum uric acid, eGFR, TC, TG, LDL-C and cathepsin S were possible risk factors of CVD (**Table 4**). In the multivariate model, these factors were defined as covariates, while CVD was introduced as a dependent variable. Notably, after adjusting for history of hypertension, DBP, 2 h C peptide, serum uric acid, eGFR, TC and TG, the results demonstrated that alcohol consumption (odds ratio, OR = 2.984, 95% confidence interval (CI): 1.696–5.250, *P* = 0.000), BMI (OR = 1.183, 95% CI: 1.085–1.290, *P* = 0.000), PBG (OR= 1.082, 95% CI: 1.028–1.139, *P* = 0.003), 2 h insulin (OR= 1.019, 95% CI: 1.008–1.030, *P* = 0.001), LDL-C (OR= 3.600, 95% CI: 1.474–8.790, *P* = 0.005) and cathepsin S (OR= 1.055, 95% CI: 1.028–1.083, *P* = 0.000) remained significantly related to the CVD (**Table 5**).

**Table 4:** Univariate logistic regression analysis indicating possible factors associated with CVD in patients with type 2 diabetes.

Parameters	B	SE	OR	95% CI	*p* value
Age	0.010	0.011	1.010	0.989–1.032	0.350
Sex	0.295	0.224	1.343	0.865–2.086	0.188
Duration of diabetes	−0.004	0.015	0.996	0.968–1.026	0.813
Smoking	−0.222	0.276	0.801	0.466–1.376	0.422
Alcohol	0.516	0.229	1.675	1.069–2.625	0.024^*^
Hypertension	0.784	0.284	2.189	1.255–3.821	0.006^**^
BMI	0.181	0.037	1.199	1.115–1.288	0.000^***^
WC	0.000	0.013	1.000	0.975–1.025	0.983
WHR	0.210	1.849	1.234	0.033–46.211	0.910
SBP	0.010	0.006	1.010	0.998–1.021	0.091
DBP	0.028	0.010	1.028	1.008–1.049	0.006^**^
FBG	−0.009	0.034	0.991	0.927–1.059	0.787
PBG	0.047	0.020	1.049	1.008–1.091	0.020^*^
HbA1c	0.025	0.068	1.025	0.896–1.172	0.720
Fasting insulin	0.010	0.009	1.010	0.993–1.028	0.253
2 h insulin	0.021	0.004	1.022	1.013–1.031	0.000^***^
Fasting C peptide	0.050	0.048	1.052	0.958–1.155	0.290
2 h C peptide	0.042	0.017	1.042	1.008–1.078	0.015^*^
HOMA-IR	0.028	0.021	1.029	0.987–1.072	0.181
BUN	0.018	0.063	1.019	0.901–1.152	0.770
Scr	0.005	0.005	1.005	0.995–1.016	0.312
Serum uric acid	4.319	1.414	75.100	4.704–1198.996	0.002^**^
eGFR	−0.008	0.004	0.992	0.984–1.000	0.041^*^
TC	0.200	0.094	1.222	1.016–1.470	0.033^*^
TG	0.264	0.111	1.303	1.048–1.619	0.017^*^
LDL-C	0.339	0.128	1.403	1.092–1.803	0.008^**^
HDL-C	−0.355	0.319	0.701	0.375–1.311	0.266
cathepsin S	0.052	0.011	1.054	1.031–1.077	0.000^***^

P < 0.05 (*), P < 0.01 (**), P < 0.001 (***).

**Table 5:** Multivariate logistic regression analysis indicating factors independently associated with CVD in patients with type 2 diabetes.

Parameters	B	SE	OR	95% CI	*p* value
Alcohol	1.093	0.288	2.984	1.696–5.250	0.000^***^
Hypertension	0.350	0.366	1.419	0.692–2.910	0.340
BMI	0.168	0.044	1.183	1.085–1.290	0.000^***^
DBP	−0.001	0.013	0.999	0.973–1.026	0.950
PBG	0.079	0.026	1.082	1.028–1.139	0.003^**^
2 h insulin	0.019	0.005	1.019	1.008–1.030	0.001^**^
2 h C peptide	−0.007	0.020	0.993	0.955–1.033	0.724
Serum uric acid	3.407	1.953	30.172	0.656–1386.791	0.081
eGFR	−0.007	0.005	0.993	0.983–1.003	0.181
TC	−0.683	0.352	0.505	0.253–1.006	0.052
TG	−0.136	0.153	0.873	0.647–1.179	0.376
LDL-C	1.281	0.455	3.600	1.474–8.790	0.005^**^
cathepsin S	0.054	0.013	1.055	1.028–1.083	0.000^***^

P < 0.01 (**), P < 0.001 (***).

**Figure 3:**
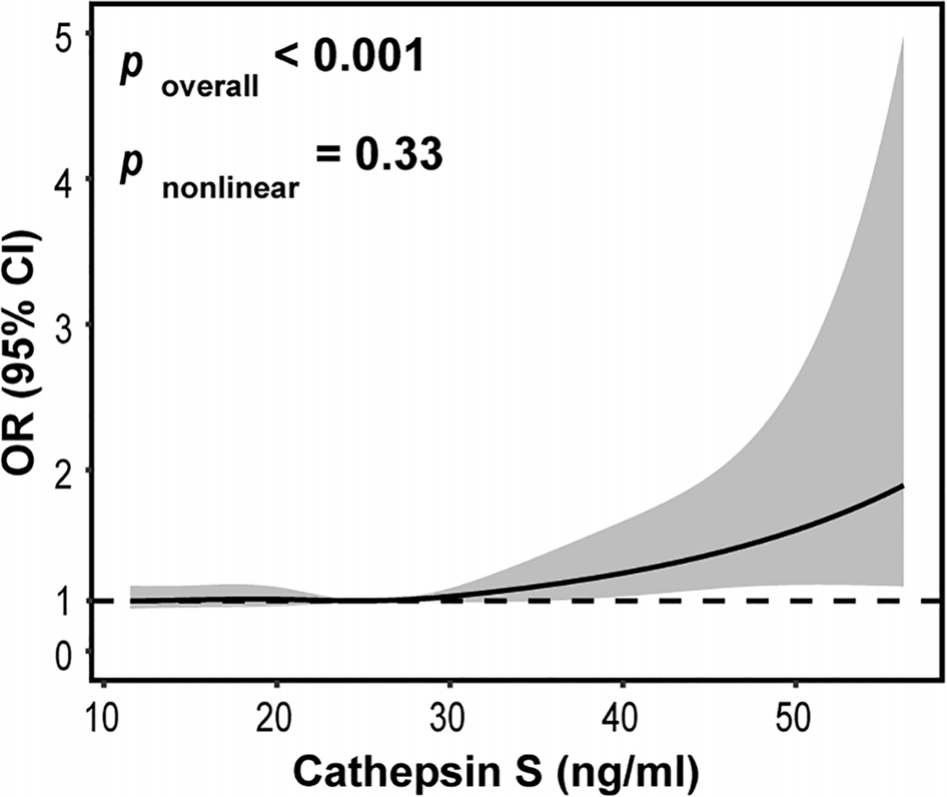
Restricted cubic spline for the association between circulating cathepsin S and CVD. The solid line represents the odds ratio (OR) and the gray area represents the 95% confidence interval (CI). Model was adjusted for age, smoking, alcohol consumption and the history of hypertension.

To further explore the potential relationship between circulating cathepsin S and CVD, we also used restricted cubic spline (RCS) model. The adjustment factors of RCS analysis for circulating cathepsin S were age, smoking, alcohol consumption and the history of hypertension. Knots of RCS were placed at the 25th, 50th, and 75th percentiles of the serum cathepsin S distribution. Interestingly, we found a significant linear association between circulating cathepsin S and CVD (*P* overall < 0.001, *P* nonlinear = 0.33). As shown in **Figure 3**, with the increase of serum cathepsin S levels, the risk of the CVD incidence also increased.

### The Diagnostic Performance of Cathepsin S for CVD

**Figure 4:**
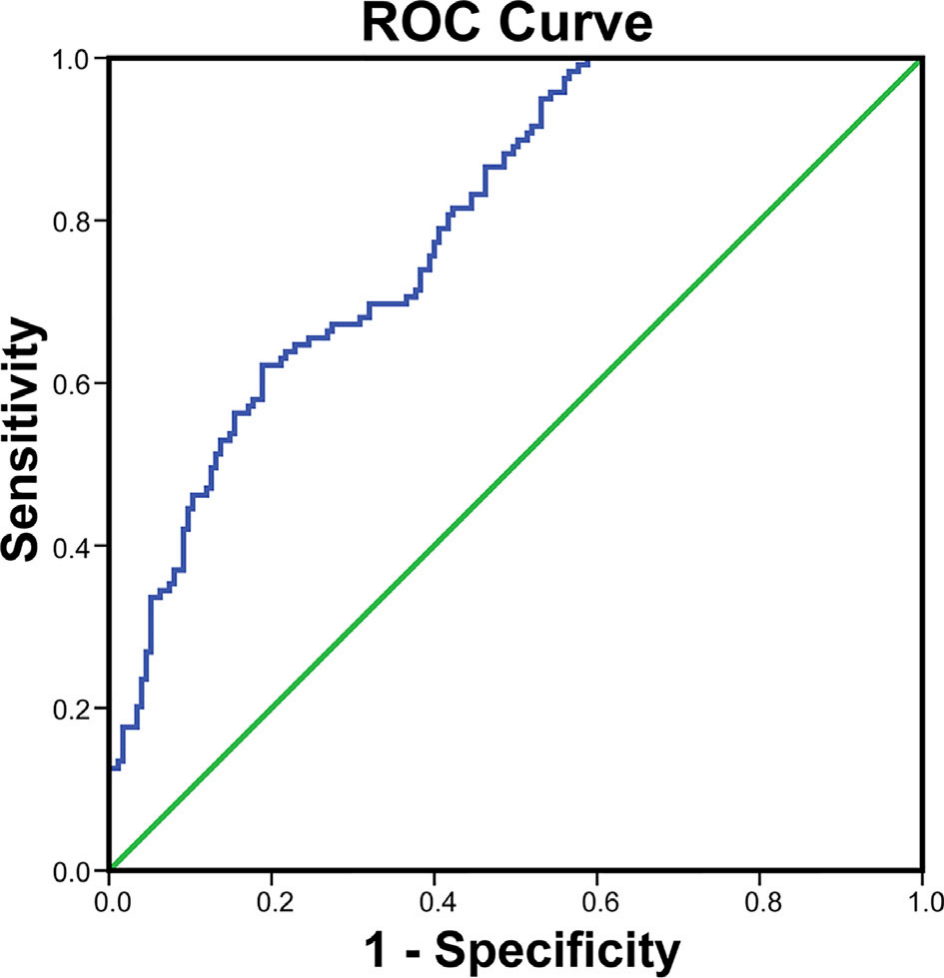
Receiver operator characteristic (ROC) curve to determine the cutoff value of cathepsin S for CVD in patients with type 2 diabetes.

To evaluate whether cathepsin S could be a diagnostic indicator for CVD, ROC analysis was used to fit the relation. As shown in **Figure 4**, cathepsin S had an area under curve (AUC) value of 0.80 (95% CI: 0.75–0.84, *P* < 0.001). And the optimal cutoff value of cathepsin was 26.28 ng/ml, with sensitivity of 62.2% and specificity of 81.1%. These results indicated that cathepsin S level may be a potential diagnostic biomarker for CVD.

## Discussion

In this study, we investigated the relationship between circulating cathepsin S and cardiovascular disease in patients with type 2 diabetes. The major finding was that levels of serum cathepsin S were substantially higher in the CVD (+) group than that in the CVD (−) one (*P* = 0.000). Also, we observed that patients with ACS had significantly higher levels of serum cathepsin S than those with SAP. And cathepsin S was associated with several cardiovascular risk factors. In addition, univariate and multivariate logistic regression analysis revealed that circulating cathepsin S was an independent risk factor for CVD (all *P* < 0.001). Meanwhile, circulating cathepsin S had a linearity relationship with the risk of CVD. Furthermore, ROC curve analysis showed that cathepsin S may be a potential diagnostic biomarker for CVD with high sensitivity and specificity.

Cathepsin S, a proteolytic enzyme, has been reported to be associated with atherosclerosis, abdominal aortic aneurysm, cancer, obesity and type 2 diabetes. Several studies found cathepsin S contributes to the formation and progression of atherosclerotic plaques and participates in the destabilization of advanced plaque (17, 18). Liu et al. reported that serum cathepsin S levels were significantly higher in atherosclerotic stenosis patients (9). Since atherosclerosis is one of the coronary artery disease, these findings are in accordance with our results. In addition, circulating cathepsin S levels were found to be elevated in the cerebral infarction individuals (19) and the abdominal aortic aneurysm subjects (20), which represent cerebral and peripheral vascular disease respectively, further supporting our discoveries. What’s more, in our study, we discovered when sub-grouped the CAD into UAP and ACS, higher serum cathepsin S levels were found in patients with ACS than those with UAP. Meanwhile, previous studies showed cathepsin S levels were highly correlated with BMI (21) and WHR (22), which are in alignment with our findings. Since SBP, DBP, TG are established cardiovascular risk factors, it is not surprising serum cathepsin S correlated with these factors. Jobs et al. revealed higher serum cathepsin S levels were associated with increased mortality risk among elderly individuals, especially related to the cardiovascular and cancer mortality (23). However, Wuopio et al. found it was cathepsin B, not cathepsin S, which was associated with an increased risk of cardiovascular events in patients with stable coronary heart disease (24). The discrepancy between age and disease entities may explain the difference. And our study adds evidence supporting the critical role of cathepsin S in predicting and diagnosing the CVD. Meanwhile, we interestingly found circulating cathepsin S had a linearity association with the risk of CVD, with the increase of cathepsin S levels, the risk of CVD incidence also increased, which have not been reported before. Furthermore, univariate and multivariate logistic regression analysis showed that serum cathepsin S as well as traditional risk factors such as alcohol status, BMI, PBG, 2 h insulin, LDL-C were independently associated with CVD. Collectively, our study indicated that serum cathepsin S levels were strongly and independently associated with CVD in patients with type 2 diabetes. It should be noted that the association between cathepsin S and CVD maybe a weak one when comparing with other diagnostic markers including alcohol status, BMI, PBG, and LDL-C. We must note that patients with CVD were under treatment with anti-hypertensive (ARBs or ACEIs) and lipid lowering drugs during the follow-up period. And it is reported that statin medications or angiotensin antagonist usages may reduce serum cathepsin S levels (25, 26). Previous studies found that a few of the intermediate endpoints (HDL-C and HbA1c) generally considered to be reliable failed to predict clinical benefit when following pharmacological intervention (27). Thus, the weak correlation coefficient might be in part due to the pharmacological interventions. Further investigations are needed to elucidate this issue.

Although we cannot establish causality in this study, there are several potential mechanisms that may explain the association between circulating cathepsin S and CVD in type 2 diabetes patients. Cathepsin S, as a proteolytic enzyme, could degenerate the extracellular matrix protein (ECM) and thereby contributes to the cardiovascular pathogenesis (13). Active cathepsin S was found in the extracts of human atherosclerotic lesions (28), and cathepsin S expression in macrophages colocalized with areas of elastin fragmentation (17). Meanwhile, cathepsin S may act like matrix metalloproteinases (MMPs) and release adhesion molecules such as vascular cell adhesion molecule-1 (VCAM-1), intercellular cell adhesion molecule-1 (ICAM-1) (29), because studies revealed deficiency of cathepsin S reduced the circulating adhesion molecules in high-cholesterol diet induced atherosclerosis mouse model (18). Additionally, cathepsin S aggravates foam cell formation by degrading low-density lipoprotein and reducing cholesterol efflux from macrophages (30). Meanwhile, cathepsin S was found to be involved in the inflammatory process (31) and in diseases such as diabetes, obesity and cancer, and these factors are associated with increased risks of CVD.

Vascular disease which affects macrovasculature is an important cause of morbidity and mortality in patients with diabetes. A simple and noninvasive tool for predicting and diagnosing CVD in type 2 diabetes patients is of great need. However, numerous studies have only focused on the relationship between cathepsin S and atherosclerosis, cathepsin S and coronary artery disease, cathepsin S and metabolic syndrome, or cathepsin S and diabetes (8, 9, 32–36), little is known about the direct relationship between cathepsin S and CVD in the context of type 2 diabetes. Liu et al. reported that serum cathepsin S levels were increased not only in patients with atherosclerosis, but also in those with type 2 diabetes (9), yet, patients enrolled in these cohorts were restricted to one disease. Thus, the relationship between cathepsin S and CVD in patients with type 2 diabetes has not been unveiled so far. To the best of our knowledge, this is the first study to assess the relationship between cathepsin S and CVD based on a community investigation in Chinese individuals with type 2 diabetes. Meanwhile, this study provides a novel insight into the potential role of cathepsin S in the diagnosis of CVD in patients with type 2 diabetes. Moreover, in this study, apart from patients diagnosed with coronary artery disease (CAD), we also enrolled those with cerebral and peripheral vascular disease, expanding the disease variety. Interestingly, our study provides further evidence that serum cathepsin S had a linearity relationship with the risk of CVD, which has not been reported before. In addition, the optimal cutoff value of cathepsin S in detecting CVD in patients with type 2 diabetes has not been suggested to date. In this study, we investigated this relationship and analyzed the optimal cut-off value of cathepsin S, which indicated an optimal cut-off of 26.28 ng/ml with 62.2% sensitivity and 81.1% specificity respectively. And compared with other cysteine cathepsin family members, cathepsin S has unique ability to retain activity at the neutral pH (37), which highlights it as an ideal target for disease diagnosis.

There are several limitations in our study. First, this was a cross-sectional study, so we could not assess the causal relationship between the cathepsin S and CVD, which needs further prospective studies to illustrate. Second, we had a relatively small study population. Although the serum samples were preserved at below −80°C, we cannot preclude the possibility that proteins degrade in some rare cases, which may lead to measurement deviations. In addition, for ethical reasons, we considered it was too problematic to use non-drug-interventions or stop-drug-interventions during follow-up period, so the patients continued to receive anti-hypertensive, anti-platelet, anti-hyperlipidemia and anti-hyperglycemic treatment. Statin medication has been implicated in the lysosomal enzyme activity and release, which may affect serum cathepsin S levels (25). And it is reported that the aforementioned drugs exert vascular remodeling and atherosclerotic regression effect in animal and humans (38–41). In our study, patients in the CVD (+) group tended to have higher rates of anti-hypertensive (ARBs or ACEIs) (40.2%) and lipid lowering drug usages (56.1%) than the CVD (−) one, which might influence our observation results. Finally, the underlying mechanisms of the role of cathepsin S in CVD under diabetic condition remains to be explored.

## Conclusion

Taken together, the present study found that circulating cathepsin S was significantly higher in the CVD (+) group than that in the CVD (−) one among type 2 diabetes patients. The increased serum cathepsin S levels were associated with increased risks of CVD, even after adjusting for potential confounders. Thus, cathepsin S might be a potential diagnostic biomarker for CVD.

## Data Availability Statement

The original contributions presented in the study are included in the article/supplementary material. Further inquiries can be directed to the corresponding authors.

## Ethics Statement

The studies involving human participants were reviewed and approved by The Institutional Review Broad of Huashan Hospital, Fudan University. The patients/participants provided their written informed consent to participate in this study.

## Author Contributions

YJ, ZY, and XW designed the study. JS, BL, YY, JW, and RH collected and organized the data. YJ, JS, BL, and WZ did the statistical analysis. YJ wrote the first draft of the paper. BL, WZ, YY, JW, and RH supervised the whole study and gave critical comments to this manuscript. ZY and XW revised the primary manuscript. All authors contributed to the article and approved the submitted version.

## Funding

This work was funded by the grants from the National Natural Science Foundation of China (81370953, 81370936, 81873645, and 81873853), the Science and Technology Commission of Shanghai Municipality (16140901200, 16PJ1401700, and 18140902100).

## Conflict of Interest

The authors declare that the research was conducted in the absence of any commercial or financial relationships that could be construed as a potential conflict of interest.

## Abbreviation

CVD, cardiovascular disease; CAD, coronary artery disease; SAP, stable angina pectoris; ACS, acute coronary syndrome; UAP, unstable angina pectoris; AMI, acute myocardial infarction; BMI, body mass index; WC, waist circumference; WHR, waist-hip ratio; SBP systolic blood pressure; DBP, diastolic blood pressure; FBG, fasting blood glucose; PBG, postprandial blood glucose; HOMA-IR, homeostatic model assessment of insulin resistance; HbA1c, glycated hemoglobin A1c; TC, total cholesterol; TG, triglycerides; LDL-C, low density lipoprotein-cholesterol; HDL-C, high density lipoprotein-cholesterol; BUN, blood urea nitrogen; Scr, serum creatinine; eGFR, estimated glomerular filtration rate; ARBs, angiotensin II receptor blockers; ACEIs, angiotensin converting enzyme inhibitors; CCB, calcium channel blockers.
